# GM-CSF Promotes the Expansion and Differentiation of Cord Blood Myeloid-Derived Suppressor Cells, Which Attenuate Xenogeneic Graft-vs.-Host Disease

**DOI:** 10.3389/fimmu.2019.00183

**Published:** 2019-02-26

**Authors:** Mi-Young Park, Bang-Geul Lim, Su-Yeon Kim, Hyun-Jung Sohn, Sueon Kim, Tai-Gyu Kim

**Affiliations:** ^1^Catholic Hematopoietic Stem Cell Bank, The Catholic University of Korea, Seoul, South Korea; ^2^Department of Microbiology, College of Medicine, The Catholic University of Korea, Seoul, South Korea; ^3^Department of Biomedicine and Health Sciences, The Catholic University of Korea, Seoul, South Korea

**Keywords:** myeloid-derived suppressor cells (MDSCs), umbilical cord blood (CB), CD34^+^ cells, recombinant GM-CSF and SCF cytokine combinations (GM-CSF/SCF), immunosuppressive function, xenogeneic graft-vs.-host disease (GVHD), FoxP3^+^ regulatory T cells (Treg)

## Abstract

Myeloid-derived suppressor cells (MDSCs) are increased in tumor patients. Studies have shown generation of MDSCs from human peripheral blood mononuclear cells (PBMCs) by various cytokine combinations. However, large scale expansion of human MDSCs has not been demonstrated or applied in clinic settings. We investigated which cytokine combinations among GM-CSF/SCF, G-CSF/SCF, or M-CSF/SCF efficiently expand and differentiate human MDSCs following culture CD34^+^ cells of umbilical cord blood (CB). GM-CSF/SCF showed the greatest expansion of MDSCs. Up to 10^8^ MDSCs (HLA-DR^low^CD11b^+^CD33^+^) could be produced from 1 unit of CB following 6 weeks of continuous culture. MDSCs produced from culture of CD34^+^ cells with GM-CSF/SCF for 6 weeks had the greatest suppressive function of T cell proliferation and had the highest expression of immunosuppressive molecules including iNOS, arginase 1 and IDO compared to those differentiated with G-CSF/SCF or M-CSF/SCF. MDSCs secreted IL-10, TGB-β, and VEGF. The infusion of expanded MDSCs significantly prolonged the survival and decreased the GVHD score in a NSG xenogeneic model of GVHD. Injected MDSCs increased IL-10 and TGF-β but decreased the level of TNF-α and IL-6 in the serum of treated mice. Notably, FoxP3 expressing regulatory T (Treg) cells were increased while IFN-γ (Th1) and IL-17 (Th17) producing T cells were decreased in the spleen of MDSC treated mice compared to untreated GVHD mice. Our results demonstrate that human MDSCs are generated from CB CD34^+^ cells using GM-CSF/SCF. These MDSCs exhibited potent immunosuppressive function, suggesting that they are useable as a treatment for inflammatory diseases such as GVHD.

## Introduction

Allogeneic hematopoietic stem cell transplantation (allo-HSCT) is a potentially curative procedure for a variety of malignancies and benign hematological disorders (1). The therapeutic benefit of allo-HSC is mediated by T cells of donor origin in blood that kill the leukemia, known as graft vs. leukemia response (2). However, the widespread use of allo-HSCT is limited by risks such as graft rejection and graft-vs.-host disease (3, 4). GVHD remains the major cause of mortality and morbidity after the allo-HSCT and is caused by a reaction of allo-reactive donor T cells to incompatible human leukocyte antigens (HLA) of the recipient. The ensuing proliferation/activation of other immune cells lead to a variety of injuries to host tissues caused by the release of inflammatory cytokines. The immunosuppressing drugs (ISDs) such as cyclosporine, tacrolimus, and steroids are used to modulate the adaptive immune system through blocking T cell activation or depletion of T cells. Further, all ISDs carry the risk of serious infections. Depletion of T cells from donor allograft can improve the survival rates for patients and decrease the incidence of acute GVHD. However, this comes at the risk of graft failure, reduced GVL activity, and increased incidence of leukemic relapse. Therefore, a new therapeutic approach is needed to optimize GVL and minimize GVHD. Recent strategies have introduced the infusion of newly identified immune suppressor cells such as mesenchymal stromal cells (MSCs) (5–7), regulatory T (Treg) cells (8–10), and myeloid-derived suppressor cells (MDSCs) (11–13) to take advantage of their immunoregulatory properties in order to prevent GVHD.

Umbilical cord blood (CB) is considered the most plentiful reservoir of regenerative cells for a large number of clinical applications (14, 15). CB has been used as a source to generate regulatory immune cells. Use of CB-derived MSCs had been investigated *in vitro*, in animal models, and in early stage clinical trials for cardiovascular diseases (16), as well as liver diseases, neurological deficits, immune system diseases, lung, and kidney injury (17). The use of CB-derived, *ex vivo*-expanded Treg or MSCs is currently being evaluated as one strategy to prevent GVHD. Their adoptive transfer has been associated with improved survival in mice (5, 8). Recently, others have reported that the fibrocytic MDSCs from CB expressed indoleamine dioxygenase (IDO), and promoted tolerance via Treg-cell expansion (18).

MDSCs are known to accumulate in the peripheral blood, lymphoid organ, spleen, and tumor sites in cancer, infection, chronic inflammation, transplantation, and autoimmunity (19, 20). These cells inhibit T cell proliferation following stimulation with allo-antigens. Importantly, MDSCs mediate the chemotaxis and activation of Treg cells *in vivo* (21, 22). The CD14^+^HLA-DR^low/neg^ monocytic MDSCs are significantly expanded in the peripheral blood of acute GVHD patients who received allo-HSCT, resulting in T cells dysfunction and GVHD inhibition (23, 24). The factors triggering MDSC expansion and activation are well-studied in tumor models, including cytokines such as IL-1β, IL-6, IL-10, and IL-13, growth factor such as SCF, VEGF, GM-CSF, G-CSF, and M-CSF, as well as calcium binding pro-inflammatory proteins such as S100A8, S100A9, cyclooxygenase-2, and prostaglandin E2 (25, 26). However, it is not known how to expand human MDSCs to a large scale enough to make their use feasible for clinical applications.

Here, we demonstrate that the combination of GM-CSF/SCF is the most potent enhancer to expand and differentiate functional MDSCs from human cord blood compared to G-CSF/SCF or M-CSF/SCF. We further show that adoptive transfer of CB-derived MDSCs ameliorate GVHD in a xenogeneic NSG mouse model.

## Materials and Methods

### Subjects and Isolation of Cells With the MACS System

The use of human peripheral blood mononuclear cells (PBMCs) and human umbilical cord blood (CB) were approved by the institutional review board of the College of Medicine, Catholic University of Korea, Seoul, Republic of Korea, respectively (permit No. MC16SNSI0001, MC15TISE0023, MC17TNSI0002). Human peripheral blood samples were obtained from healthy donors, and mononuclear cells were isolated by Ficoll-Hypaque (Amersham Pharmacia Biotech Inc., Piscataway, NJ, USA) density gradient centrifugation. After density separation, CD14^+^ monocytes and CD4^+^ T cells were isolated with the magnetic cell-sorting (MACS) system (Miltenyi Biotec, Bergisch Gladbach, Germany), using anti-CD14 and anti-CD4 antibodies, respectively, conjugated to magnetic MicroBeads (Miltenyi Biotec) according to the manufacturer's instructions.

### Generation of Human MDSCs

Human CB was provided from the Catholic Hematopoietic Stem Cell Bank after written informed consent given by normal full-term pregnant women. For MDSCs generation, isolated CD34^+^ cells (Miltenyi Biotec, Bergisch Gladbach, Germany) were cultured in a 48-well plate (BD Falcon, Bedford, MA) at 1 × 10^5^ cells/ well with 1 ml of IMDM containing 10% FBS (Gibco, Grand Island, NY, United States), 10% penicillin–streptomycin (100 U/ml; Lonza Walkersville, MD, United States), 2 mM L-glutamine (Lonza Walkersville) (10% complete medium), 100 ng/ml human GM-CSF (300–03, PeproTech, Rocky Hill, NJ, United States), 100 ng/ml human G-CSF (300–23, PeproTech), or 100 ng/ml human M-CSF (300–25, PeproTech) and 50 ng/ml human SCF (300–07, PeproTech). After incubation for 7 days, the cells were removed from the 48 well plate and centrifuged at 1,300 rpm for 5 min. After one wash with serum free IMDM, the cells were cultured for 2 weeks and media was changed every 7 days. From weeks 4–6, the cells were cultured at a higher density (5 × 10^5^ cells/well). Media was changed every 7 days throughout 6 weeks of the culture.

### Production of HCMV pp65 mRNA by *in vitro* Transcription

The sequences encoding full-length pp65 were cloned into the pcDNA3 vector (Invitrogen, Grand Island, NY, United States). The pcDNA3-pp65 were linearized with Sma I restriction enzyme and purified using phenol/chloroform extraction and ethanol precipitation. In *vitro* transcription of recombinant pp65 from the linearized plasmids was conducted by using T7 RNA polymerase of Ambion mRNA T7 Ultra Kit (Life Technologies) according to the manufacturer's instructions.

### Generation of Monocyte Derived DCs and pp65 mRNA Electroporation

Immature DCs (iDCs) were generated from CD14^+^ monocytes of human PBMCs by culturing them with the CD14^+^ cells were cultured with human GM-CSF (100 ng/ml; PeproTech) and IL-4 (100 ng/ml; PeproTech) in 10% complete RPMI 1640 medium (Lonza Walkersville) for 6 days. Media was changed every 2 days. On day 6, 5 × 10^6^ iDCs were resuspended with 200 μl OptiMEM without phenol red (Invitrogen Life Technologies, Grand Island, NY). The cells were transfected with 20 μg pp65 mRNA to a 2 mm cuvette with a single wave pulse (300 V and 500 μs) by using a Gene pulser (BTX, San Diego, CA). The electroporated iDCs were matured at 1 × 10^6^ cells/ml in 37°C, 5% CO_2_ for 24 h using a maturation cocktail containing 100 ng/mL IL-4, 100 ng/mL GM-CSF, 10 ng/mL TNF-α (PeproTech), 10 ng/mL IL-6 (PeproTech), and 10 ng/mL IL-1β (PeproTech).

### Flow Cytometric Analysis

All samples were incubated with anti-CD16/CD32 to block Fc receptor binding on ice for 20 min and then stained with the indicated anti-human antibodies. The expression of MDSCs was assessed by staining with monoclonal antibodies specific for surface markers including CD33, CD11b, HLA-DR, CD14, CD15, CD11c, CD13, HLA-ABC, CD45, CD40, CD80, CD86, CD83, PDL-1, CCR2, CCR5, CCR7, CD62L, E-Cadherin, CXCR4, and ICAM-1 (Supplemental Table 1)

For intracellular staining of pStat, pAkt and pmTOR, the MDSCs were lysed and fixed using BD Phosflow Lyse/Fix buffer for 10 min at 37°C. Cells were then permeabilized in BD Phosflow Perm Buffer III on ice for 30 min. For intracellular staining of iNOS, IDO, arginase 1, MPO, and FoxP3 (For Treg analysis, anti-human CD4 PerCP-Cy5.5 and anti-human CD25 APC were already stained), the MDSCs were fixed in BD Cytofix buffer and then permeabilized in BD Cytoperm buffer. Cells were then washed twice in the stain buffer and stained on ice for 30 min with monoclonal antibodies specific for pStat1, pStat3, pStat6, pAkt, pmTOR, iNOS, IDO, arginase 1, MPO, FoxP3 (Supplemental Table 1). The compensation bead (UltraComp eBeads Catalog No. 01-2222, Invitrogen) were used to avoid the spillover of fluorescence conjugated antibodies. All samples were acquired on a BD LSR Fortessa and then analyzed using FlowJo 9.2.1.

### Cell Sorting

Cells cultured with GM-CSF/SCF were harvested at 3 weeks post culture and stained with anti-human CD33-FITC antibody and anti-human CD11b-PE antibody. CD33^+^ CD11b^+^ and CD33^+^ CD11b^−^ were sorted by a FACS Aria sorter (BD Biosciences).

### Suppression Assay

To evaluate suppressive activity of MDSCs, PBMCs were labeled with 5 μmol/ml CFSE (Invitrogen, Cat.No.C34554) and activated with Dynabead Human T-Activator anti-CD3 and anti-CD28 (0.5 μg/mL, Gibco, Cat.No.11131D) microbeads. MDSCs were added to parallel cultures at ratio of 1:1, 1:0.5, and 1:0.25 (PBMCs: MDSCs) ratio. After 6 days of co-culture, cells were harvested and stained with anti-human CD3 PE-Cy7, CD4 APC, and CD8 efluor450 antibodies. The cells were analyzed on a FACSCanto II flow cytometer (BD Biosciences, San Jose, CA). Data were analyzed using ModFit LT software (Verity Software House Inc., Topsham, ME, United States).

### Treg Induction by MDSCs

The CD4^+^ T cells were isolated from healthy adult PBMCs. 1 × 10^6^ CD4^+^ T cells were stimulated with Dynabead Human T-Activator anti-CD3 and anti-CD28 (0.5 μg/mL) microbeads in the presence or absence of 2 × 10^6^ MDSCs at a 1:2 (CD4^+^ T cells: MDSCs) ratio in 12 well plates for 3 days. After 3 days of co-culture, the cells were harvested and Treg induction was determined by flow cytometric analysis.

### 3H-Thymidine Mixed Lymphocytes Reaction (MLR) Assay

Mature DCs (1 × 10^4^) were mixed with responder CD4^+^ T cells (1 × 10^5^) in 96 well plates with or without MDSCs (1 × 10^4^). After 5 days culture, cell were pulsed with 1 μCi [^3^H]thymidine/well for 18 hrs and harvested using a Packard filtermate cell harvester (Packard Instruments, Meriden, CT). Specific [^3^H]thymidine incorporation into genomic DNA was determined using a Packard TopCount NXT.

### ELISPOT Assay

To measure the effect of MDSCs on antigen-specific T cell responses, ELISPOT assay was performed using DCs electroporated with mRNA encoding human cytomegalovirus (HCMV) pp65 antigen according to the manufacturer's instructions (BD ELISPOT assay kit; BD Biosciences) as described previously (27). Because HCMV infects between 60 and 90% of adults and then it remains latent under control of immune system, T cells specific for various HCMV antigens are present in a high frquency, mainly in response to the HCMV pp65 antigen. Therefore, we used mononuclear cells from a healthy volunteer donor that has been shown to have a high T cell immune response to the HCMV pp65 antigen. It has been established in our previous study that DCs electroporated with mRNA encoding HCMV pp65 antigens present antigen to T cells through HLA class I and class II molecules after antigens processing, so T cell responses to whole antigens are measured rather than specific HLA-restricted antigen epitopes (28).

Briefly, anti-human IFN-γ antibody was pre-coated in a 96 well-microplates for 24 h at 37°C. pp65 RNA-electroporated mature DCs were added to a 96-well-microplates at a concentration of 1 × 10^4^ cells/well in 10% complete RPMI 1640. 1 × 10^5^ autologuous CD4^+^ T cells were then added to the well as a stimulator in the presence or absence MDSCs (1 × 10^4^). After incubation for 24 h, the cells were removed and the plates were washed three times with the wash buffer and the PBS-Tween buffer, respectively. The wells were added with the biotinylated antibody for human IFN-γ and incubated for 2 h at room temperature. The plates were washed with PBS-Tween buffer, and then incubated with streptavidin-HRP for 1 h at room temperature. The washing of well was repeated, and then the 100 μl AEC substrate and H_2_O_2_ were added to the well. After development of the spots, the reaction was stopped with addition of distilled water. Plates were dried for overnight. The spots number of IFN-γ-secreting cells was quantitated with an automatic an AID-ELISPOT reader (AID Diagnostika GmbH, Strassberg, Germany).

### Assessment of Pro-inflammatory Cytokine and Anti-inflammatory Cytokine by ELISA

ELISA was performed according to the manufacturer's instructions (R&D Systems). Human IL-17, IFN-γ, transforming growth factor (TGF)-β, IL-10, and vascular endothelial growth factor (VEGF) were measured in the cell culture supernatant. Whole blood collected from mouse was centrifuged at 400 × g for 10 min, and the serum was analyzed for the levels of human IL-10, TGF-β, tumor necrosis factor (TNF)-α, and IL-6. The plates were then read using an ELISA microtiter plate autoreader at 450 nm (Molecular Devices, Sunnyvale, CA, United States).

### Xenogeneic GVHD Mouse Model

NSG (NOD.Cg-Prkdcscid Il2rgtm1Wjl/SzJ, 6 weeks old) male mice were purchased from the Jackson Laboratory (Bar Harbor, ME, USA) and acclimated in pathogen-free animal facilities for 2 weeks before the experiments. All animal work was approved in advance by the Institutional Animal Care and Use Committee, College of Medicine, Catholic University of Korea. Recipient (8 weeks old) mice were irradiated with 200 cGy by Mevatron MXE-2 instrument (Siemens, New York, NY, United States) at day −1 and received 1 × 10^6^ human PBMCs intravenously (IV) at day 0 to induce xenogeneic GVHD mouse models. The control did not receive human PBMCs or irradiation. To examine the effect of MDSCs in a xenogenic GVHD model, MDSCs (1, 2.5 and 5 × 10^6^ cells) were infused into the tail vein on days 21 and 24. Survival after PBMCs transplantation was observed daily, and the grade of clinical GVHD was recorded every other day using a scoring system on the basis of weight loss, posture, physical activity, fur texture, and skin integrity. We evaluated GVHD according to overall survival rate until day 200. Each group was consisted of total eight mice and three mice were sacrificed at day 60 for FACS analysis and ELISA, respectively.

### Multi-Cytokine Membrane Array

Serum collected from GVHD mice or MDSCs mice was analyzed using the human cytokine array (Proteome Profiler Array Human Cytokine, R&D Systems, ARY005). The array analysis was performed according to the manufacturer's instructions. Those cytokine levels were captured by exposure to the X-ray films and quantified by densitometry using Image J software.

### Statistical Analysis

Data were analyzed for statistical significance using Prism version 6.0 (GraphPad, San Diego, CA). Student's *t*-test or ANOVA was used to calculate the significance between groups. Differences in animal survival (Kaplan Meier curves) were analyzed by log-rank test. Results were considered statistically significant if *p* ≤ 0.05.

## Results

### Expansion of Cord Blood CD34^+^ Cells and Differentiation Into MDSCs

**Figure 1:**
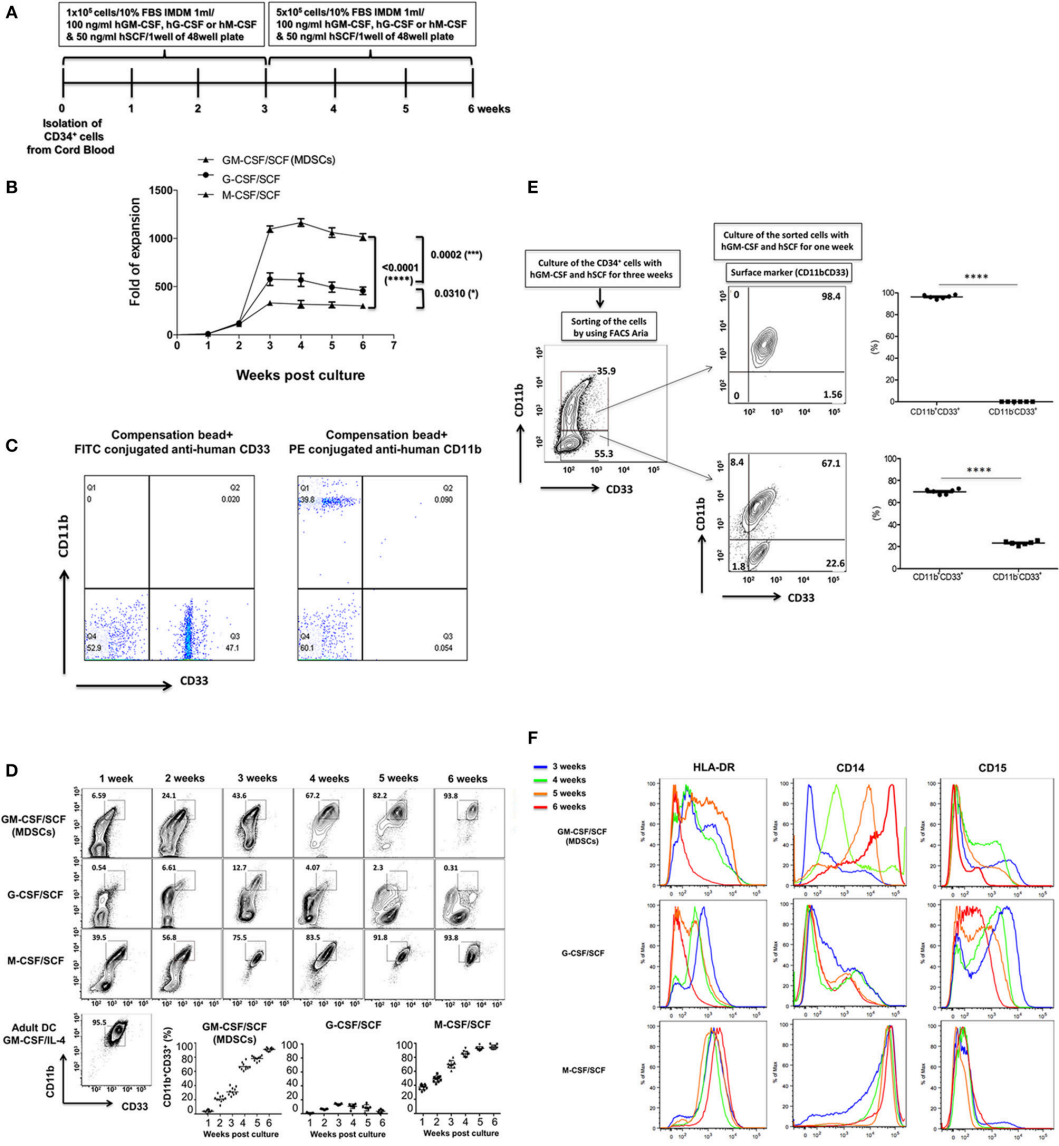
Expansion of CB CD34^+^ cells according to cytokine combinations and differentiation into MDSCs. **(A)** The scheme of cell expansion or differentiation from CB CD34^+^ cells. **(B)** Fold expansion by different cytokine combinations. The culture medium was replaced with each cytokine combinations every week. **(C)** The FITC conjugated anti-human CD33 were compensated with compensation bead to avoid spillover with PE conjugated anti-human CD11b. **(D)** Expression of CD11b^+^CD33 ^+^. The cells were stained with fluorochrome conjugated anti-human CD33 (FITC) and aniti-human CD11b (PE) every week. **(E)** The CD34^+^ cells were cultured with human GM-CSF (100 ng/ml) and human SCF (50 ng/ml) for 3 weeks and the cells were stained like **(D)**. The stained cells were sorted by FACS Aria and the sorted cells (CD11b^+^CD33b^+^ vs. CD11b^−^CD33^+^) were cultured with human GM-CSF (100ng/ml) and human SCF (50ng/ml) for a further 1 week. After one week, the sorted cells were stained like **(D)** and analyzed by flow cytometry. **(F)** Expression of HLA-DR, CD14 and CD15. The cells were stained with efluor450-conjugated anti-human HLA-DR, PE-Cy7 anti-human CD14 and APC anti-human CD15 from 3 to 6 weeks, respectively and analyzed by flow cytometry. These experiments were reproduced in 10 individuals (from **B** to **D** and **F**) and 6 individuals **(E)**.

To generate MDSCs from cord blood, 1 × 10^5^ CD34^+^ umbilical cord blood cells were plated in 48 well plates. Recombinant human cytokines such as GM-CSF/SCF, G-CSF/ SCF or M-CSF/SCF were added weekly and cultured for an additional 3 weeks. After 3 weeks, the 5 × 10^5^ cells per well were plated in 48 well plates for 3 weeks (Figure 1A). The cells were most effectively expanded by the combination of GM-CSF/SCF (MDSCs). Up to 10^8^ MDSCs could be generated from 1 unit of cord blood. This is more than a thousand folds (1,104 ± 70.30) increase. The expansion of cells cultured with G-CSF/SCF or M-CSF/SCF were 500 (514 ± 42.62) and 300 (317 ± 39.27) fold, respectively (Figure 1B). After 3 weeks of culture, expansion of CD34^+^ cells reached stagnation in the three different groups. CD34^+^ cells were continuously differentiated until 6 weeks (Figure 1D). MDSCs from GM-CSF/SCF or M-CSF/SCF expressed CD11b^+^CD33^+^ (90% of cells positive for these markers) at 6 weeks. Only 10% of cells from G-CSF/SCF cultures expressed CD11b^+^CD33^+^ at 3 weeks. The CD11b^+^CD33^+^ almost disappeared by 6weeks following G-CSF/SCF cultures (Table 1).

**Table 1:** Characterization of cultured cells for 6 weeks.

	**GM-CSF/SCF**	**G-CSF/SCF**	**M-CSF/SCF**	
Level of HLA-DR	low	low	high	Figure 1F
CD11b^+^CD33^+^	high	dim	high	Figure 1D
Monocyte (CD14):Granulocytes(CD15) ratio	9: 1 (M-MDSCs)	2: 8	10:0	Figure 1F
Level of Arginase 1 and IDO	+++	+	++	Figure 3A
Suppressive activity	+++	++	+	Figure 4A
Treg induction	+++	++	++	Figure 4B

*Typical nomenclature of human myeloid derived suppressor cells (MDSCs): HLA-DR^negative or low^, CD11b^+^CD33^+^, CD14^+^: Monocytic MDSCs (M-MDSCs). HLA-DR^negative or low^, CD11b^+^CD33^+^, CD15^+^: Granulocytic MDSCs (G-MDSCs)*.

To further define the differentiation of MDSCs from GM-CSF/SCF, CD11b^+^CD33^+^ and CD11b^−^CD33^+^ cells at 3 weeks were sorted via FACS, and then cultured with GM-CSF/SCF for a further 1 week (Figure 1E). The sorted CD11b^−^CD33^+^ cells changed to a phenotype that expressed more than 69.92 (69.92 ± 0.92)% of CD11b^+^ CD33^+^ and the phenotype of the sorted CD11b^+^ CD33^+^ cells remained unchanged. Therefore, this result demonstrates that GM-CSF/SCF induces differentiation from the CD11b^−^CD33^+^ cells to the CD11b^+^CD33^+^ cells. Next, we evaluated the expression of HLA-DR, CD14, and CD15 in cells cultured with GM-CSF/SCF, G-CSF/SCF, or M-CSF/SCF at 3 weeks and 6 weeks (Figure 1F). The expression of HLA-DR in the cells cultured with GM-CSF/SCF or G-CSF/SCF was gradually decreased and became low, whereas cells cultured with M-CSF/SCF highly expressed HLA-DR at 3 and 6 weeks, respectively. From 3 to 6 weeks, the expression of CD14 were increased in the cells cultured with GM-CSF/SCF or M-CSF/SCF whereas that of CD15 were decreased (Figure 1F, Table 1). At 6 weeks of culture, cells cultured with GM-CSF/SCF showed the phenotypic markers of monocytic MDSCs; HLA-DR^low^, CD11b^+^CD33^+^, CD14^+^, CD15^−^. These results suggest that GM-CSF/SCF among cytokine combinations most efficiently induces the cell expansion during 3 weeks of culturing with at low cell concentration, and the differentiation to MDSC progresses during the remaining 3 weeks of culturing at 5 fold higher cell concentration.

### Lineage Markers and Immunophenotypes of MDSCs

**Figure 2:**
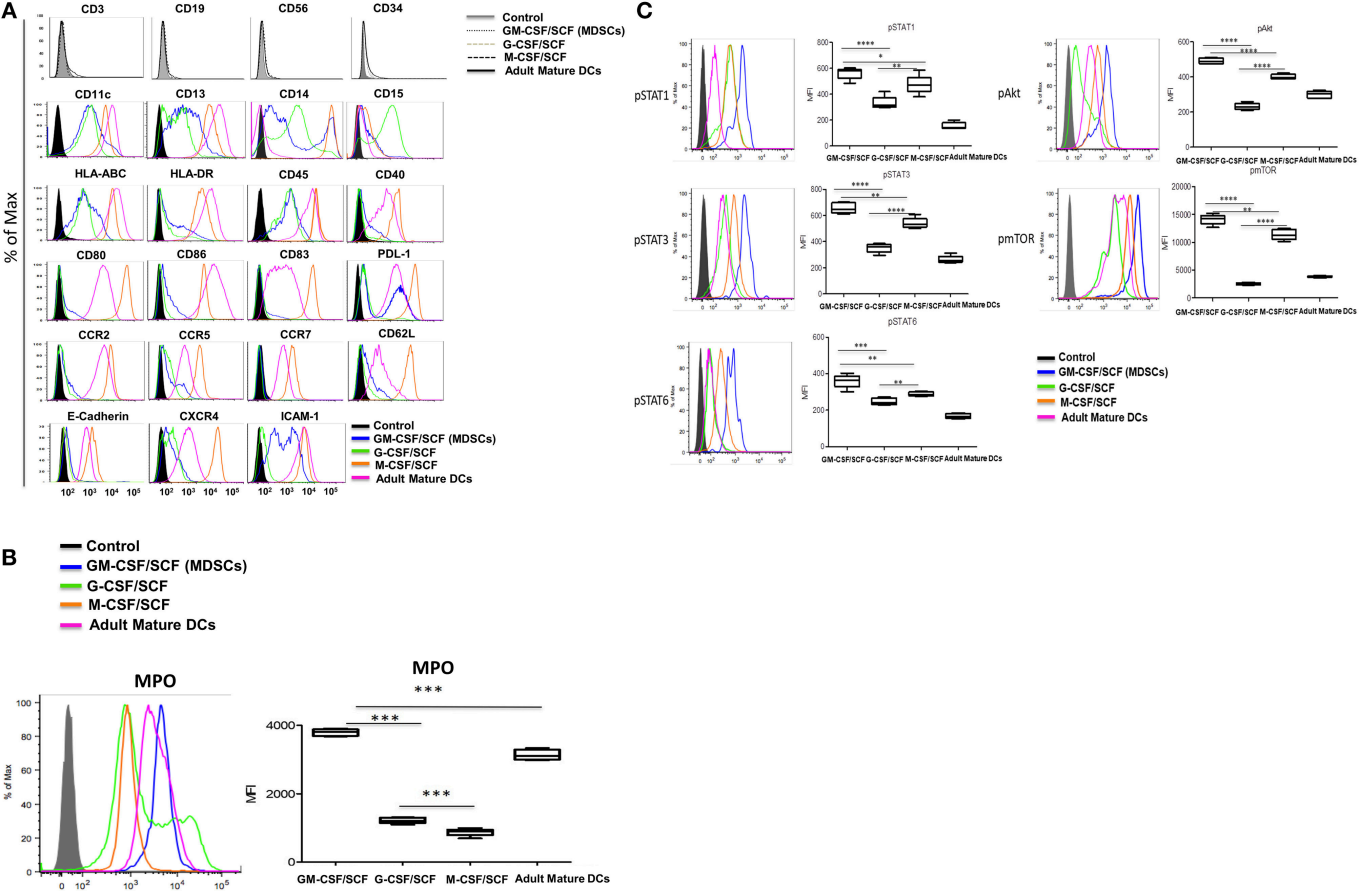
Characterization of cultured MDSCs. **(A)** FACS profiles of cells cultured with human GM-CSF, G-CSF, or M-CSF (100 ng/ml) and human SCF (50 ng/ml) for 6 weeks. Blue histogram lines represent the GM-CSCF/SCF (MDSCs), light green histogram lines the cells cultured G-CSF/SCF, orange histogram lines the cells cultured M-CSF/SCF and pink histogram lines represent the adult mature DCs. **(B)** The cells were intracellular stained with anti-human MPO antibody and analyzed by flow cytometry. **(C)** The cells were lysed and fixed in a single step using Phosflow Lyse/Fix buffer for 10 min at 37°C. Cells were then permeabilized in Phosflow Perm Buffer for 30 min on ice. Cells were then washed twice in the stain Buffer and stained with fluorochrome conjugated anti-Stat (AF647), anti-Stat3 (PE), anti-Stat6 (PerCP-Cy5.5), anti-Akt (PE) and anti-mTOR (PerCP-efluor718) antibodies for 30 min on ice. Cells were washed twice in the stain buffer. All samples were acquired on a BD LSR Fortessa and then analyzed using FlowJo 9.2.1. Data are mean ± S.E.M. of three independent experiments. *p < 0.05, **p < 0.01, ***p < 0.001, ****p < 0.0001.

We performed immunophenotypic characterization of MDSCs following 6 weeks of GM-CSF/SCF, G-CSF/SCF, and M-CSF/SCF culture. Expression of surface markers was examined using flow cytometry and adult monocyte derived mature DCs (mature DCs) were compared using as controls, since a variety of immune molecules are strongly expressed. The lineage and differentiation markers showed that the cells were lineage-negative for CD3, CD19, and CD56 but were uniformly positive for myeloid associate marker for CD11b (Figure 1C), CD11c, and CD13 (Figure 2A).

Cell surface markers associated with immune functions are shown in Figure 2A (Table 2). The expression of HLA-ABC was positive in all cells cultured with the three different cytokine combinations. In contrast, the expression of HLA-DR was negative in G-CSF/SCF, expressed as low level in GM-CSF/SCF and high in M-CSF/SCF cultured cells (Table 1). The expression of costimulatory molecules CD86 and CD40 were low and CD80 and CD83 were negative in GM-CSF/SCF cultured cells. The chemokine receptors CCR2, CCR5, CD62L, and CXCR4 were expressed at low levels in both GM-CSF/SCF and G-CSF/SCF cultured cells. However, the M-CSF/SCF cultured cells showed the high expression of chemokine receptors. The cells cultured with M-CSF/SCF expressed E-cadherin (mediated cell–cell adhesion) at high levels, but the expression on cells cultured with GM-CSF/SCF and G-CSF/SCF were negative. ICAM-1 was not expressed in G-CSF/SCF cultured cells. The GM-CSF/SCF and M-CSF/SCF cultured cells showed the high expression for PDL-1. PDL-1 expression on G-CSF/SCF cultured cells were low. Myeloperoxidase (MPO) is a heme protein synthesized during myeloid differentiation. GM-CSF/SCF cultured cells showed a significantly higher level of MPO activity than G-CSF/SCF and M-CSF/SCF (Figure 2B).

**Table 2:** Immunophenotypes of cultured cells for 6 weeks (MFI).

	**GM-CSF/SCF (MDSCs)**	**G-CSF/SCF**	**M-CSF/SCF**	**Adult mature DCs**
CD11c	10^3^	10^3^	10^4^	10^4^
CD13	10^3^	10^3^	10^4^	10^4^
CD14	10^5^	10^3^	10^5^	10^2^
CD15	10^2^	10^3^	10^2^	10^2^
HLA-ABC	10^3^	10^3^	10^4^	10^4^
HLA-DR	10^2^	10^2^	10^4^	10^4^
CD40	10^1^	10^1^	10^3^	10^3^
CD80	10^1^	10^1^	10^4^	10^3^
CD86	10^1^	10^1^	10^3^	10^4^
CD83	10^1^	10^1^	10^4^	10^3^
PDL-1	10^3^	10^2^	10^4^	10^1^
CCR2	10^1^	10^1^	10^4^	10^4^
CCR5	10^2^	10^2^	10^4^	10^3^
CCR7	10^1^	10^1^	10^4^	10^3^
CD62L	10^2^	10^1^	10^4^	10^3^
E-Cadherin	10^1^	10^1^	10^3^	10^3^
CXCR4	10^2^	10^2^	10^4^	10^3^
ICAM-1	10^3^	10^2^	10^4^	10^4^

### Signal Molecules of MDSCs

We next tested for expression of molecules (phosphorylated-(p) Stat1, pStat3, pStat6, pmTOR, and pAkt) related to MDSCs signaling pathways (Figure 2C). The GM-CSF/SCF cultured cells showed the highest expression levels of pStat1, pStat3, pStat6, pmTOR, and pAkt protein. The G-CSF/SCF showed the lowest expression in these signaling except pStat1 which showed similar expression level compared with M-CSF/SCF. These results demonstrate that cells cultured with GM-CSF/SCF expressed signaling molecules associated with MDSCs. Taken together, our phenotypic data suggests that CB CD34^+^ cells cultured with GM-CSF/SCF or G-CSF/SCF were consistent with MDSCs. Cells cultured with M-CSF/SCF were consistent with monocytes.

### Immune Suppressive Molecules in MDSCs

**Figure 3:**
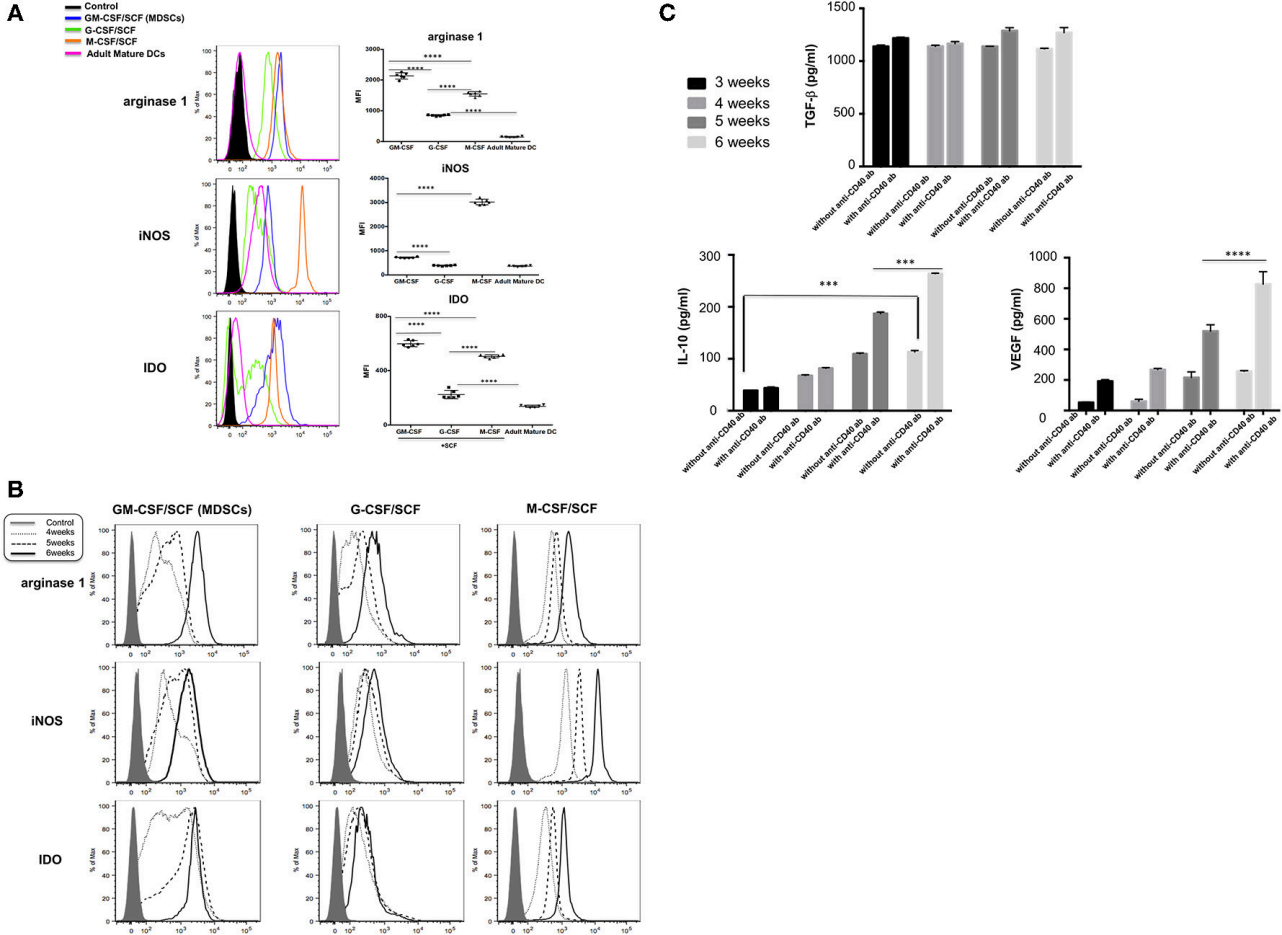
MDSCs express immune suppressive molecules. **(A)** The cells cultured with each cytokine combinations for 6 weeks or **(B)** The cells cultured with each cytokine combinations from 4 to 6 weeks were fixed in Cytofix buffer for 10 min at 37°C and then permeabilized in Cytoperm buffer for 30 min on ice. Cells were then washed twice in the stain buffer and stained with FITC anti-iNOS antibody, PE anti-IDO antibody, and PerCP-Cy5.5 anti-arginase 1 antibody 30 min on ice. Cells were washed twice in the stain buffer and acquired on a BD LSR Fortessa. **(C)** The cells cultured with GM-CSF/SCF were stimulated with or without anti-CD40 antibody for 48 h and human VEGF, TGF-β and IL-10 were compared in culture supernatants between 3 and 6weeks. Data are mean ± S.E.M. of three independent experiments, each performed in triplicate. ***p < 0.001, ****p < 0.0001.

Expression of inhibitory molecules such as Arginase, IDO, and inducible nitric oxide synthase (iNOS) in MDSCs was investigated by intracellular stain. Expression of arginase 1 and IDO was significantly higher in MDSCs generated by GM-CSF/SCF culture compared to those from M-CSF/SCF culture (Table 1). The expression of iNOS in GM-CSF/SCF culture was significantly lower following culture with M-CSF/SCF. The expression of these inhibitory molecules was lowest in G-CSF/SCF culture. In addition, the expression of arginase 1 and IDO was negative in the mature DCs but iNOS were intermediately expressed in the mature DCs (Figure 3A). The results showed that the level of arginase 1, iNOS, and IDO gradually increased from 4 to 6 weeks of culture with GM-CSF/SCF (Figure 3B).

The sustained and induced secretion of immunosuppressive cytokines such as human VEGF, TGF-β and IL-10 was measured in culture supernatants (3 weeks through 6 weeks) of CB CD34^+^ cells differentiated with GM-CSF/SCF to become MDSCs (Figure 3C). Stimulation of GM-CSF/SCF derived MDSCs (GM-CSF/SCF MDSCs) with anti-CD40 antibody resulted in markedly elevated secretions of IL-10 and VEGF. The TGF-β was produced to high levels regardless of stimulation. The expressions of these inhibitory molecules were indicative of the immunosuppressive functions of GM-CSF/SCF derived MDSCs.

### Immune Suppressive Functions on T Cells of MDSCs *in vitro*

**Figure 4:**
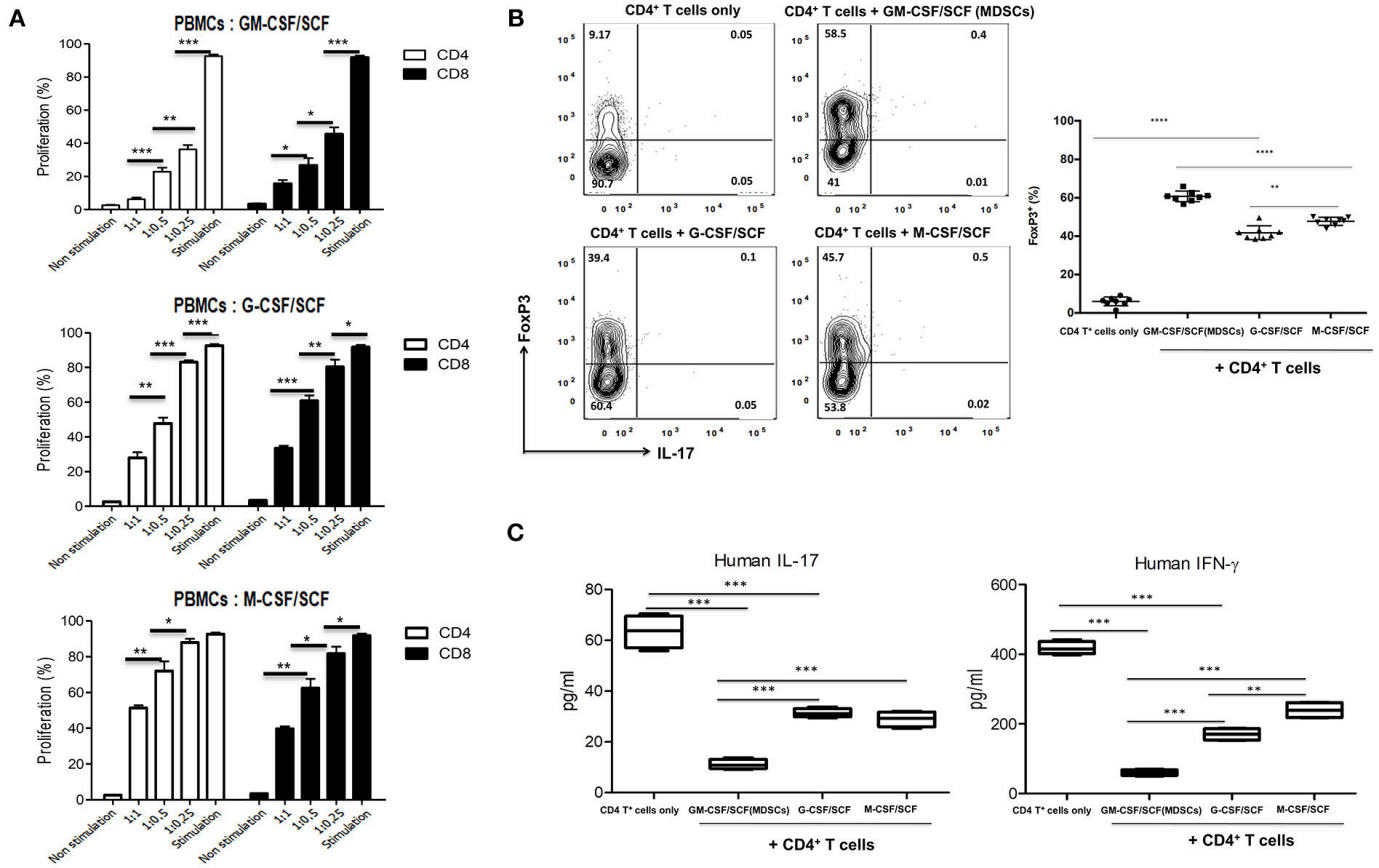
MDSCs have suppressive effect for T cells and induce a polarization of helper T cells. **(A)** Healthy adult PBMCs were labeled with CFSE and stimulated with Dynabead Human T-Activator CD3 and CD28 in the presence of MDSCs at a 1:1, 1:0.5, and 1:0.25 (PBMCs: MDSCs) ratio. After 6 days, cells were then harvested, stained with anti-human CD3 (PE-Cy7), anti-human CD4 (APC) and anti-human CD8 (efluor450) antibodies. The cells were analyzed by FACSCanto II device. **(B)** CD4^+^ T cells were isolated from healthy adult PBMCs and 1 × 10^6^ CD4^+^ T cells were stimulated with 0.5 μg/mL Dynabead Human T-Activator CD3 and CD28 in the presence or absence of 2 × 10^6^ MDSCs at a 1:2 (CD4^+^ T cells: MDSCs) ratio in 12 well plates for 3 days. After 3 days, the cells were stained with fluorochrome-conjugated anti-human CD3 (PE-Cy7), anti-human CD4 (FITC), anti-human CD25 (APC), anti-human IL-17 (PerCP-Cy5.5) and anti-human FoxP3 (PE) antibodies. **(C)** Human IL-17 and IFN-γ was measured in the culture supernatants of **(B)**. Data are mean ± S.E.M. of three independent experiments, each performed in triplicate. *p < 0.05, **p < 0.01, ***p < 0.001, ****p < 0.0001.

To define whether the MDSCs exert suppressive function on T cells *in vitro*, adult human PBMCs were labeled with CFSE and then stimulated with anti-CD3 and anti-CD28 microbeads in the presence or absence of MDSCs. After 6 days of co-culture, T-cell proliferation was assessed by measuring CFSE intensity within CD4^+^ and CD8^+^ subsets (Figure 4A). GM-CSF/SCF MDSCs showed the strongest suppressive effect on both CD4^+^ T cells (6.3% [range, 3.3–9.2%]), and CD8^+^ T cells (15.3% [range, 9.4–20.0%]) at 1:1 (PBMC: MDSC) ratio (Table 1). M-CSF/SCF generated cells exhibited the weakest suppressive ability.

To determine if cytokine generated MDSCs could alter the polarization of helper T cells, the cells were co-cultured with adult naïve CD4^+^ T cells for 3 days. We then measured the expression of FoxP3 (Figure 4B) in CD4^+^ gated Treg cells. We also measured the expression of IL-17 and IFN-γ (Figure 4C) in culture supernatants. The GM-CSF/SCF MDSCs resulted in a highest frequency of FoxP3^+^ Treg cells (58.5 ± 3.3 %) compared with G-CSF/SCF (39.4 ± 5.2 %) and M-CSF/SCF (45.7 ± 2.1 %) (Table 1). By contrast, MDSCs from all groups did not secrete the IL-17 (Figure 4B). The GM-CSF/SCF MDSCs mediated the most potent suppression of human IL-17 and IFN-γ production. The G-CSF/SCF exhibited decreased production of human IFN-γ but human IL-17 was not significant between G-CSF/SCF and M-CSF/SCF (Figure 4C). The GM-CSF/SCF MDSCs most effectively inhibited T cell proliferation and production of inflammatory cytokines and led to the greatest production of FoxP3^+^ Treg cells.

### Inhibition of T Cells Proliferation and Antigen-Specific T Cell Responses by MDSCs

**Figure 5:**
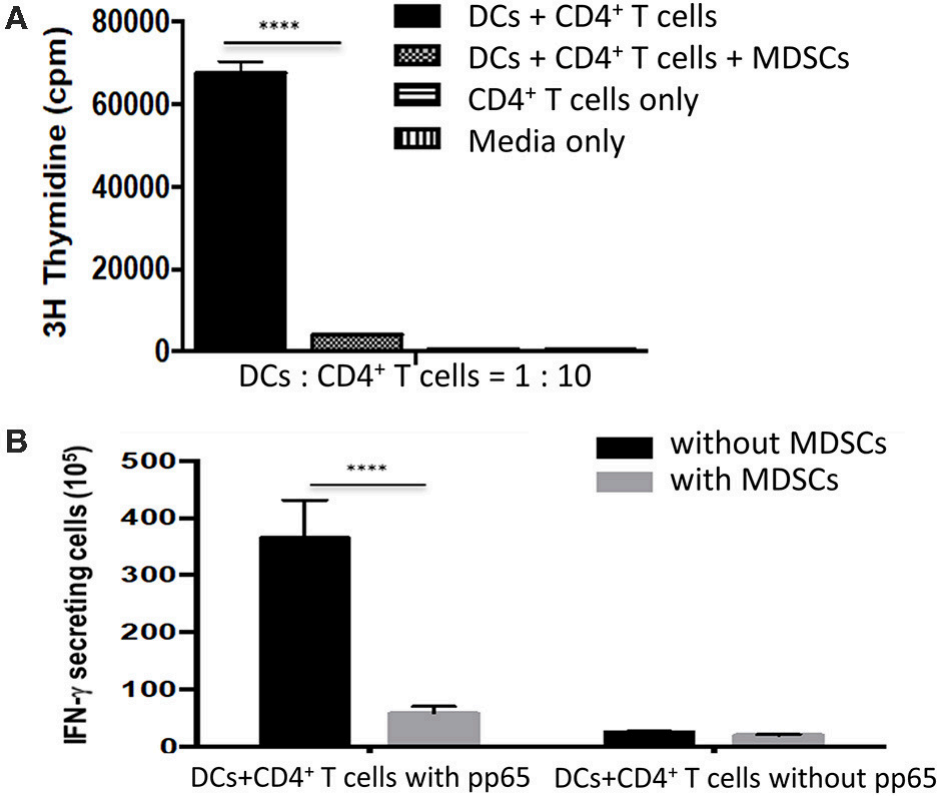
MDSCs effectively suppress the proliferation of CD4^+^ T cells and antigen-specific CD4^+^ T cells responses. **(A)** CD4^+^ T cells isolated from healthy adult PBMCs were used as stimulators from three different donors. Adult monocyte derived mature DCs (1 × 10^4^ cells) were mixed with the autologous CD4^+^ T cells (1 × 10^5^ cells) in the presence or absence 1 × 10^4^ MDSCs. The cells were cultured for 5 days and 1 μCi [^3^H]thymidine was added to the wells for 18 hr before harvesting. The incorporation of [^3^H]thymidine was determined using a liquid β-scintillation counter. **(B)** The HCMV pp65 RNA-electroporated mature DCs were added to a 96-well microplate at a concentration of 1 x 10^4^ cells/well in 10% complete RPMI 1640. 1 × 10^5^ autologous CD4^+^ T cells were then added to the well as a stimulator in the presence or absence MDSCs (1 × 10^4^). An ELISPOT assay for human IFN-γ was conducted according to the manufacturer's protocol. Data are mean ± S.E.M. of three independent experiments, each performed in triplicate. ****p < 0.0001.

GM-CSF/SCF derived MDSCs was chosen for further study because they exhibited the greatest expression of immune suppressive molecules (Figures 3A,C) and greatest immune suppressive function (Figure 4A). To address the suppressive capacity of T cells proliferation by MDSCs *in vitro*, the mature DCs were co-cultured with adult naïve CD4^+^ T cells in the presence or absence GM-CSF/SCF MDSCs. The reactivity of T cells was assessed based on the incorporation of [3H]thymidine. GM-CSF/SCF MDSCs profoundly reduced the ability to prime responses of T cells (Figure 5A). To examine the inhibition of antigen-specific T cell responses by MDSCs, the pp65 mRNA were electroporated into adult monocyte derived immature DCs. The DCs were matured and co-cultured with adult naïve CD4^+^ T cells in the presence or absence GM-CSF/SCF MDSCs. The MDSCs markedly inhibited (7-fold decreases) the number of pp65 specific IFN-γ secreting cells (Figure 5B).

### Inhibition of GVHD by MDSCs in the Xenogeneic Mouse Model

**Figure 6:**
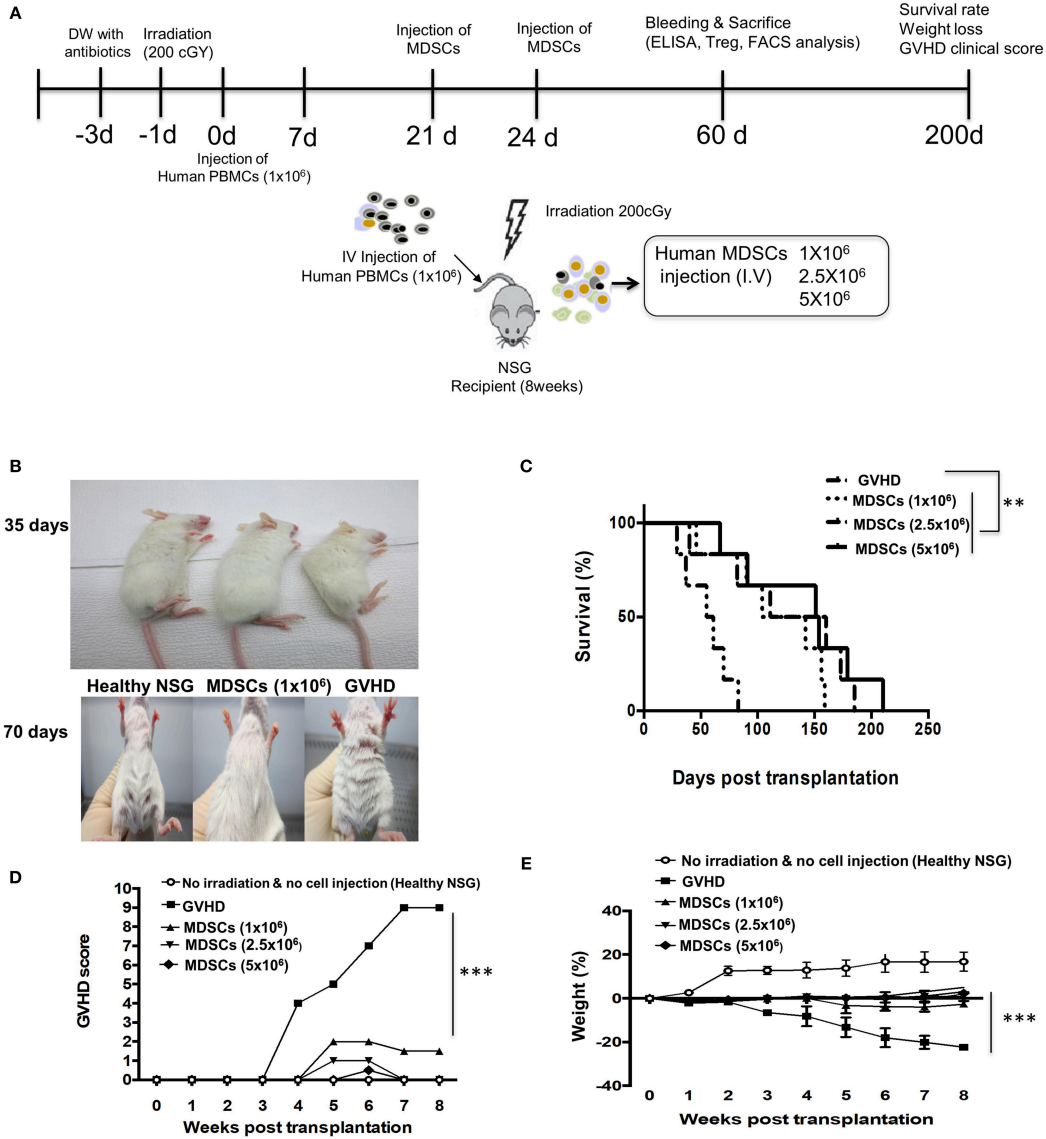
MDSCs infusion ameliorates xenogeneic GVHD. **(A)** Experimetnal scheme. NSG mice were administered with/without sublethal irradiation (200 cGy) at day−1 and 1 × 10^6^ human PBMCs were infused at day 0. The NSG mice were administered either 1 × 10^6^, 2.5 × 10^6^, 5 × 10^6^ MDSCs (MDSCs mice) or PBMCs only (GVHD mice) at day 21 and 24. **(B)** Condition of fur: MDSCs mice vs. GVHD mice at day 35 and 70. **(C)** Overall survival. At a median of 100 days after PBMCs infusion, MDSCs mice were > 60% as compared with <0% for GVHD mice (GVHD vs. MDSCs 1 × 10^6^ = 0.0070, GVHD vs. MDSCs 2.5 × 10^6^ = 0.0156, GVHD vs. MDSCs 5 × 10^6^ = 0.0059; log-rank test; *n* = 5 mice/group). Data represent one experiment of two independent experiments (MDSCs vs. GVHD **p < 0.01; log-rank test; n = 5 mice/group). **(D)** The degree of GVHD was recorded daily using a scoring system that summed changes in five clinical parameters: weight loss, posture, physical activity, fur texture, and skin integrity. The MDSCs mice showed lower GVHD score (MDSCs vs. GVHD ***p < 0.001). **(E)** The weight was measured every other day and the MDSCs treatment attenuates a weight loss (MDSCs vs. GVHD ***p < 0.001). Data are mean ± S.E.M. of two independent experiments.

Lethal irradiated NSG mice were given human PBMCs to induce the fatal GVHD. In some mice, three doses of GM-CSF/SCF MDSCs were injected intravenously on days 21 and 24 (Figure 6A). The GVHD mice not given MDSCs showed hunched back on day 35 and loss of fur on day 70 (Figure 6B). Infusion of MDSCs significantly decreased GVHD scores in a dose dependent manner (Figure 6D) and inhibited the rapid decrease in body weight (Figure 6E) in comparison to control GVHD mice. The MDSCs significantly prolonged survival compared to control GVHD mice (GVHD vs. MDSCs 1 × 10^6^ = 0.0070, GVHD vs. MDSCs 2.5 × 10^6^ = 0.0156, GVHD vs. MDSCs 5 × 10^6^ = 0.0059) (Figure 6C). On day 100, all GVHD control mice died, but more than 50 % of the mice given MDSCs survived. Increased MDSCs mediated survival was not cell dose dependent. These results show that CB GM-CSF/SCF derived MDSCs can ameliorate fatal GVHD in the xenogeneic mouse model.

### Change in Anti-inflammatory and Pro-inflammatory Cytokines and Induction of FoxP3^+^ Treg Cells via MDSCs Infusion in the Xenogeneic Mouse Model

**Figure 7:**
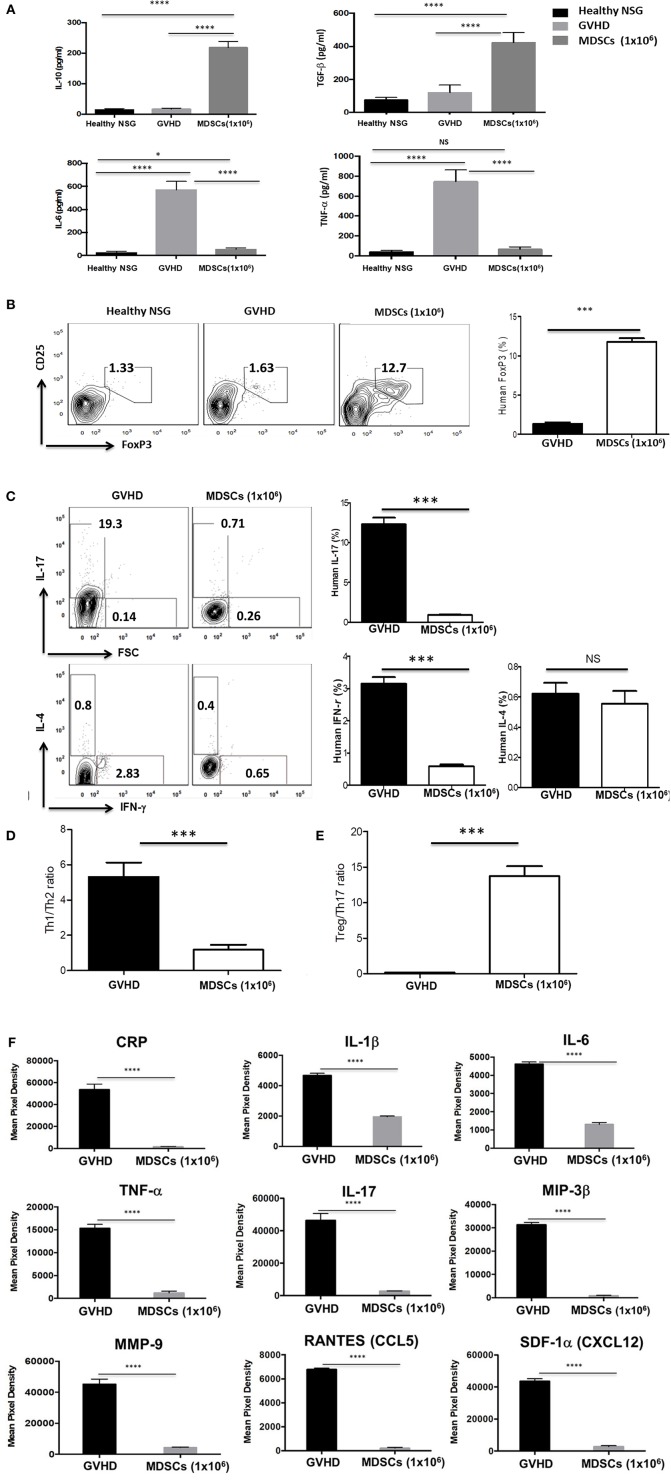
MDSCs infusion increases induction of FoxP3^+^ Treg cells and anti-inflammatory cytokine and decreases pro-inflammatory cytokine production in xenogeneic GVHD model. The mice were bled and sacrificed at day 60 post human PBMCs infusion and **(A)** the human cytokine levels in the serum were measured by ELISA kits. **(B-C)** Splenocytes were stained with fluorochrome-conjugated anti-human CD3 (PE-Cy7), anti-human CD4 (FITC) and anti-human CD25 (APC) and then intracellular stained with anti-human Foxp3 (PE), anti-human IL-17 (PerCP-Cy5.5), anti-human IFN-γ (Pacific Blue) and anti-human IL-4 (APC-Cy7) antibodies using fixation and permeabilization buffer. CD3^+^CD4^+^ T cells gated contour plots were presented. **(D)** The ratio of Th1/Th2 on CD3^+^CD4^+^ T cells. The ratio was calculated as the ratio of IL-4^+^ CD3^+^CD4^+^ T cells divided by the percentage of IFN-γ^+^ CD3^+^CD4^+^ T cells. **(E)** The ratio of Treg/Th17 on CD3^+^CD4^+^ T cells. The ratio was calculated as the ratio of IL-17^+^ CD3^+^CD4^+^ T cells divided by the percentage of CD25^+^FoxP3^+^CD3^+^CD4^+^ T cells. **(F)** Serum cytokine levels were estimated semi-quantitatively with a multi-cytokine (human) membrane array. The data were presented a decrease in the pro-inflammatory cytokine levels, including C reactive protein (CRP), IL-1β, IL-6, TNF-α, IL-17, macrophage inflammatory protein-3β (MIP-3β), matrix metallopeptidase 9 (MMP9), Regulated on Activation Normal T cell Expressed and Secreted (RANTES) and stromal cell-derived factor-1α (SDF-1α) in the MDSCs mice compared with GVHD mice (*n* = 3 mice/group). Data are mean ± S.E.M. of three independent experiments, each performed in triplicate. *p < 0.05, ***p < 0.001, ****p < 0.0001.

Mice were bled at 60 days post human PBMCs transplantation. Cytokine levels in the serum were measured by ELISA (Figure 7A). Serum levels of IL-6 and TNF-α were significantly increased in control GVHD mice, whereas IL-10 and TGF-β were similarly low in control GVHD mice compared to normal NSG mice. In contrast, serum levels of IL-10 and TGF-β in mice given 1 × 10^6^ GM-CSF/SCF MDSCs were significantly increased compared to those in normal NSG or control GVHD mice. IL-6 and TNF-α were reduced in mice given 1 × 10^6^ MDSCs compared to control GVHD mice but similar to normal NSG mice. Cytokine levels were further estimated semi-quantitatively via a multi-cytokine membrane array (Figure 7F). Mice given 1 × 10^6^ MDSCs showed lower serum levels of C reactive protein (CRP), IL-1β, IL-6, TNF-α, IL-17, macrophage inflammatory protein-3β (MIP-3β), matrix metallopeptidase 9 (MMP9), Regulated on Activation, Normal T Cell Expressed and Secreted (RANTES) and stromal cell-derived factor-1α (SDF-1α) compared to control GVHD mice.

To explore the mechanism of reduction of GVHD severity caused by Th subsets, the expression of FoxP3 and CD25 for Treg cells (Figure 7B), IFN-γ for Th1 cells, IL-4 for Th2 cells and IL-17 for Th17 cells were measured by intracellular staining assay (Figure 7C). Splenocytes from mice given 1 × 10^6^ MDSCs showed increased frequencies (11.8 % ± 0.44 %) of human FoxP3^+^ Treg cells and decreased frequencies of IL-17 (0.88 % ± 0.075 %) and IFN-γ (0.58 % ± 0.061 %) producing cells compared with those of control GVHD mice. The frequencies of CD4^+^ T cells expressing human IL-4 were very low and there was no difference between control GVHD mice and mice given 1 × 10^6^ MDSCs. The Treg/Th17 ratio (Figure 7E) was significantly higher and Th1/Th2 ratio (Figure 7D) was significantly lower in mice given 1 × 10^6^ MDSCs compared to control GVHD mice. There was no significant difference in anti-inflammatory and pro-inflammatory cytokines observed between the cell number of MDSCs administered (Figures 7A–C; data not shown).

These results show that MDSCs had a protective effect against GVHD by increasing FoxP3^+^ Treg cells *in vivo*, altering the balance among Th1, Th2, and Th17 cells and inhibiting the inflammatory responses.

## Discussion

Although efforts have been undertaken to expand MDSCs, the number of cells were limited to 3 × 10^7^ in mice (29–33). MDSCs are not typically present in healthy individuals but soluble immune modulatory factors induce the expansion of MDSCs from normal human peripheral blood mononuclear cells (34). Others have shown that CD33^+^ MDSCs with potent suppressive capacity can be generated *in vitro* by GM-CSF and IL-6, and secondarily by GM-CSF + IL-1β, PGE2, TNF-α, or VEGF. CB CD34^+^ cells cultured with GM-CSF and G-CSF for 4 days were differentiated into fibrocytic MDSCs. The cells expressed the phenotypic markers of MDSCs, DCs, and fibrocyte, and induced Treg cells by increasing of IDO expression (18). In this study, MDSCs cultured with GM-CSF/SCF expanded up to 10^8^ cells/ 1 unit of cord blood (CB) and revealed the most potent influence of expansion among three different cytokine combinations. It has been reported that the concentration of GM-CSF was related to regulation between immune suppression of MDSCs and immune stimulation of mature DCs (35). SCF plays an important role in MDSCs expansion of tumor bearing mice and suppression of tumor-infiltrating T cells. In addition, SCF receptor (ckit)–SCF interaction promoted a development of tumor and Treg (32).

Here, we characterized human MDSCs generated over 6 weeks from CB CD34^+^ cells using GM-CSF/SCF. The MDSCs showed phenotypic markers of human monocytic MDSCs; HLA-DR^low^, CD11b^+^ CD33^+^, CD14^+^. CD80, CD83, and CD86 were not expressed in GM-CSF/SCF MDSCs or cells cultured with G-CSF/SCF (G-CSF/SCF). It has been reported that low levels of GM-CSF promote myeloid cell viability in culture and expanded CD33^+^ cells (34, 36). GM-CSF/SCF have been used for generation of tolerogenic dendritic cells from CD34^+^ cells of cord blood which were expanded up to 10^8^ that expressed CD11C^+^ CD11b^+^ CD13^+^, CD80^low^ CD86^low^, and CD83 ^low^ (37). In our study, culture of CB CD34 cells with G-CSF/SCF led to expansion of cells by low expression of HLA-DR, small increase (10%) of CD11b^+^ CD33^+^. GM-CSF/SCF differentiated cells expressed MPO. These results corroborate that MDSCs were successfully generated by coculture of CB CD34^+^ cells with GM-CSF/SCF (38).

GM-CSF/SCF MDSCs had the strongest suppressive capacity to inhibit proliferation of T cells. The MDSCs increased the frequencies of FoxP3^+^ Treg cells and remarkably inhibited the generation of Th1 and Th17 cells compared with myeloid cells from G-CSF/SCF or M-CSF/SCF cultures. Furthermore, GM-CSF/SCF MDSCs inhibited the proliferation of CD4^+^ T cells and the secretion of IFN-γ by antigen-specific T cells. Bone marrow MDSCs prevent GVHD in an arginase 1–dependent manner that is up-regulated by addition of interleukin-13 (11). Under GVHD inflammatory condition, MDSCs rapidly lose their suppressive function and their potential to inhibit GVHD lethality (12). Indeed, the infusion of GM-CSF/SCF MDSCs augmented the survival and reduced GVHD lethality such as recovery of weight and GVHD score. In the mice given MDSCs, serum concentrations of most inflammatory cytokines were decreased, while IL-10 and TGF-β were increased. GM-CSF/SCF MDSCs showed an inhibitory effect on Th1 and Th17 polarization and led to increase human Foxp3^+^ Treg cells. These *in vitro* and *in vivo* results demonstrate the mechanism by which MDSCs are immunosuppressive. Consistent with these immunosuppressive functions, the expression of immunosuppressive molecules such as arginase 1, IDO, and iNOS and the secretion of immunosuppressive cytokines such as TGF-β, IL-10, and VEGF were increased in GM-CSF/SCF MDSCs in our studies. However, the pathophysiological and migration mechanisms of human MDSCs need to be further clarified to use a new therapeutic strategy for immune regulation.

Cells cultured at high cell concentration from 3 to 6 weeks stopped proliferating and progressed to differentiation instead. The level of arginase 1 and IDO were significantly higher in 6 weeks cultured cells with GM-CSF/SCF compared to those of 4 weeks. At a high cell concentration, low oxygen tensions may induce striking increase in iNOS and arginase 1 enzyme levels, suggesting a role of HIF-1a-dependent hypoxic regulation in myeloid cell-mediated T cell suppression and the differentiation of MDSCs (39). Autophagy induced by starvation or various stresses may affect the differentiation of myeloid cells (40, 41). Future studies should further investigate factors that influence this *in vitro* differentiation in order to increase understanding on the generation of MDSC in various disease status.

Preclinical studies using adoptive transfer of MDSCs have been conducted in various experimental animal models in order to treat autoimmune diseases and to inhibit the graft rejection or GVHD in organ and hematopoietic stem cell transplantation, and successful treatment effects and mechanism of action have been reported (11–13). However, clinical applications have been difficult due to the numerical limitations of MDSC. Therefore, MDSCs expanded from CB provides the possibility of being used in clinical studies to investigate the safety and therapeutic effects of adoptive transfer in these diseases.

In conclusion, the three different cytokine combinations had an obviously different influence on the differentiation and immunosuppressive functions of human MDSCs. Moreover, the GM-CSF/SCF combinations revealed to be most efficient for the generation of functionally MDSCs from CD34^+^ cells of cord blood. The human MDSCs could provide a useful strategy for the treatment of inflammatory diseases such as GVHD in the clinics.

## Ethics Statement

All animal experiments were performed according to the investigator's protocol approved in advance by the Institutional Animal Care and Use Committee, College of Medicine, Catholic University of Korea. This study involving human subjects was carried out in accordance with the recommendations of the Declaration of Helsinki. The protocol was approved by the institutional review board of the College of Medicine, Catholic University of Korea, Seoul, Republic of Korea (permit No. MC16SNSI0001, MC15TISE0023, MC17TNSI0002). All subjects gave written informed consent for sample donation in accordance with the Declaration of Helsinki.

## Author Contributions

M-YP designed research, performed experiments, analyzed data, and wrote the manuscript. B-GL performed and analyzed proliferation experiments using CFSE. S-YK performed isolation of mononuclear cells from cord blood. H-JS provided human PBMCs and reviewed the data. SK performed proliferation experiments using CFSE and edited the manuscript. T-GK designed and organized research and edited the manuscript.

### Conflict of Interest Statement

The authors declare that the research was conducted in the absence of any commercial or financial relationships that could be construed as a potential conflict of interest. The reviewer KJ and handling editor declared their shared affiliation at the time of review.

## References

[1] Gratwohl A Baldomero H Aljurf M Pasquini MC Bouzas LF Yoshimi A. Hematopoietic stem cell transplantation: a global perspective. JAMA (2010) 303:1617–24. 10.1001/jama.2010.491 20424252 PMC3219875

[2] Horowitz MM Gale RP Sondel PM Goldman JM Kersey J Kolb HJ. Graft-versus-leukemia reactions after bone marrow transplantation. Blood (1990) 75:555–62. 2297567

[3] Shlomchik WD. Graft-versus-host disease. Nat Rev Immunol. (2007) 7:340–52. 10.1038/nri2000 17438575

[4] Jacobsohn DA Vogelsang GB. Acute graft versus host disease. Orphanet J Rare Dis. (2007) 2:35. 10.1186/1750-1172-2-35 17784964 PMC2018687

[5] Jang YK Kim M Lee YH Oh W Yang YS Choi SJ. Optimization of the therapeutic efficacy of human umbilical cord blood-mesenchymal stromal cells in an NSG mouse xenograft model of graft-versus-host disease. Cytotherapy (2014) 16:298–308. 10.1016/j.jcyt.2013.10.012 24418403

[6] Yanez R Lamana ML Garcia-Castro J Colmenero I Ramirez MBueren JA. Adipose tissue-derived mesenchymal stem cells have *in vivo* immunosuppressive properties applicable for the control of the graft-versus-host disease. Stem Cells (2006) 24:2582–91. 10.1634/stemcells.2006-0228 16873762

[7] Ringden O Uzunel M Rasmusson I Remberger M Sundberg B Lonnies H. Mesenchymal stem cells for treatment of therapy-resistant graft-versus-host disease. Transplantation (2006) 81:1390–7. 10.1097/01.tp.0000214462.63943.14 16732175

[8] Parmar S Liu X Tung SS Robinson SN Rodriguez G Cooper LJ. Third-party umbilical cord blood-derived regulatory T cells prevent xenogenic graft-versus-host disease. Cytotherapy (2014) 16:90–100. 10.1016/j.jcyt.2013.07.009 24480547 PMC4124936

[9] Hess AD. Modulation of graft-versus-host disease: role of regulatory t lymphocytes. Biol Blood Marrow Transpl. (2006) 12(Suppl. 2):13–21. 10.1016/j.bbmt.2005.11.002 16399597

[10] Koreth J Matsuoka K Kim HT McDonough SM Bindra B Alyea EP III. Interleukin-2 and regulatory T cells in graft-versus-host disease. N Engl J Med. (2011) 365:2055–66. 10.1056/NEJMoa1108188 22129252 PMC3727432

[11] Highfill SL Rodriguez PC Zhou Q Goetz CA Koehn BH Veenstra R. Bone marrow myeloid-derived suppressor cells (MDSCs) inhibit graft-versus-host disease (GVHD) via an arginase-1-dependent mechanism that is up-regulated by interleukin-13. Blood (2010) 116:5738–47. 10.1182/blood-2010-06-287839 20807889 PMC3031417

[12] Koehn BH Apostolova P Haverkamp JM Miller JS McCullar V Tolar J. GVHD-associated, inflammasome-mediated loss of function in adoptively transferred myeloid-derived suppressor cells. Blood (2015) 126:1621–8. 10.1182/blood-2015-03-634691 26265697 PMC4582338

[13] Wang D Yu Y Haarberg K Fu J Kaosaard K Nagaraj S. Dynamic change and impact of myeloid-derived suppressor cells in allogeneic bone marrow transplantation in mice. Biol Blood Marrow Transl. (2013) 19:692–702. 10.1016/j.bbmt.2013.01.008 23376089 PMC4011929

[14] Gluckman E. Milestones in umbilical cord blood transplantation. Blood Rev. (2011) 25:255–9. 10.1016/j.blre.2011.06.003 21764191

[15] Roura S Pujal JM Galvez-Monton C Bayes-Genis A. The role and potential of umbilical cord blood in an era of new therapies: a review. Stem Cell Res Ther. (2015) 6:123. 10.1186/s13287-015-0113-2 26133757 PMC4489204

[16] Roura S Pujal JM Galvez-Monton C Bayes-Genis A. Impact of umbilical cord blood-derived mesenchymal stem cells on cardiovascular research. Biomed Res Int. (2015) 2015:975302. 10.1155/2015/975302 25861654 PMC4377460

[17] Li T Xia M Gao Y Chen Y Xu Y. Human umbilical cord mesenchymal stem cells: an overview of their potential in cell-based therapy. Exp Opin Biol Ther. (2015) 15:1293–306. 10.1517/14712598.2015.1051528 26067213

[18] Zoso A Mazza EM Bicciato S Mandruzzato S Bronte V Serafini P. Human fibrocytic myeloid-derived suppressor cells express IDO and promote tolerance via treg-cell expansion. Eur J Immunol. (2014) 44:3307–19. 10.1002/eji.201444522 25113564

[19] Gabrilovich DI Nagaraj S. Myeloid-derived suppressor cells as regulators of the immune system. Nat Rev Immunol. (2009) 9:162–74. 10.1038/nri2506 19197294 PMC2828349

[20] Greten TF Manns MP Korangy F. Myeloid derived suppressor cells in human diseases. Int Immunopharmacol. (2011) 11:802–7. 10.1016/j.intimp.2011.01.003 21237299 PMC3478130

[21] Lindau D Gielen P Kroesen M Wesseling P Adema GJ. The immunosuppressive tumour network: myeloid-derived suppressor cells, regulatory T cells and natural killer T cells. Immunology (2013) 138:105–15. 10.1111/imm.12036 23216602 PMC3575763

[22] Chou HS Hsieh CC Charles R Wang L Wagner T Fung JJ. Myeloid-derived suppressor cells protect islet transplants by B7-H1 mediated enhancement of T regulatory cells. Transplantation (2012) 93:272–82. 10.1097/TP.0b013e31823ffd39 22179405 PMC3267010

[23] Yin J Wang C Huang M Mao X Zhou J Zhang Y. Circulating CD14^+^ HLA-DR(-/low) myeloid-derived suppressor cells in leukemia patients with allogeneic hematopoietic stem cell transplantation: novel clinical potential strategies for the prevention and cellular therapy of graft-versus-host disease. Cancer Med. (2016) 5:1654–69. 10.1002/cam4.688 27109254 PMC4944894

[24] Mougiakakos D Jitschin R von Bahr L Poschke I Gary R Sundberg B. Immunosuppressive CD14+HLA-DRlow/neg IDO+ myeloid cells in patients following allogeneic hematopoietic stem cell transplantation. Leukemia (2013) 27:377–88. 10.1038/leu.2012.215 22828446

[25] Condamine T Gabrilovich DI. Molecular mechanisms regulating myeloid-derived suppressor cell differentiation and function. Trends Immunol. (2011) 32:19–25. 10.1016/j.it.2010.10.002 21067974 PMC3053028

[26] Gabrilovich DI Ostrand-Rosenberg S Bronte V. Coordinated regulation of myeloid cells by tumours. Nat Rev Immunol. (2012) 12:253–68. 10.1038/nri3175 22437938 PMC3587148

[27] Park MY Kim HS Woo SJ Kim CH Park JS Sohn HJ. Efficient antitumor immunity in a murine colorectal cancer model induced by CEA RNA-electroporated B cells. Eur J Immunol. (2008) 38:2106–17. 10.1002/eji.200737960 18624349

[28] Park MJ Kim EK Han JY Cho HW Sohn HJ Kim SY. Fusion of the Human Cytomegalovirus pp65 antigen with both ubiquitin and ornithine decarboxylase additively enhances antigen presentation to CD8^+^ T cells in human dendritic cells. Hum Gene Ther. (2010) 21:957–67. 10.1089/hum.2009.216 20218861

[29] Paschall AV Zhang R Qi CF Bardhan K Peng L Lu G. IFN regulatory factor 8 represses GM-CSF expression in T cells to affect myeloid cell lineage differentiation. J Immunol. (2015) 194:2369–79. 10.4049/jimmunol.1402412 25646302 PMC4340766

[30] Kohanbash G McKaveney K Sakaki M Ueda R Mintz AH Amankulor N. GM-CSF promotes the immunosuppressive activity of glioma-infiltrating myeloid cells through interleukin-4 receptor-alpha. Cancer Res. (2013) 73:6413–23. 10.1158/0008-5472.CAN-12-4124 24030977 PMC3829000

[31] Rosborough BR Castellaneta A Natarajan S Thomson AW Turnquist HR. Histone deacetylase inhibition facilitates GM-CSF-mediated expansion of myeloid-derived suppressor cells *in vitro* and *in vivo*. J Leukoc Biol. (2012) 91:701–9. 10.1189/jlb.0311119 22028329 PMC4046249

[32] Pan PY Wang GX Yin B Ozao J Ku T Divino CM. Reversion of immune tolerance in advanced malignancy: modulation of myeloid-derived suppressor cell development by blockade of stem-cell factor function. Blood (2008) 111:219–28. 10.1182/blood-2007-04-086835 17885078 PMC2200807

[33] Youn JI Kumar V Collazo M Nefedova Y Condamine T Cheng P. Epigenetic silencing of retinoblastoma gene regulates pathologic differentiation of myeloid cells in cancer. Nat Immunol. (2013) 14:211–20. 10.1038/ni.2526 23354483 PMC3578019

[34] Lechner MG Liebertz DJ Epstein AL. Characterization of cytokine-induced myeloid-derived suppressor cells from normal human peripheral blood mononuclear cells. J Immunol. (2010) 185:2273–84. 10.4049/jimmunol.1000901 20644162 PMC2923483

[35] Kusmartsev S Gabrilovich DI. Immature myeloid cells and cancer-associated immune suppression. Cancer Immunol Immunother. (2002) 51:293–8. 10.1007/s00262-002-0280-8 12111117 PMC11034227

[36] Ko JS Zea AH Rini BI Ireland JL Elson P Cohen P. Sunitinib mediates reversal of myeloid-derived suppressor cell accumulation in renal cell carcinoma patients. Clin Cancer Res. (2009) 15:2148–57. 10.1158/1078-0432.CCR-08-1332 19276286

[37] Harada Y Okada-Nakanishi Y Ueda Y Tsujitani S Saito S Fuji-Ogawa T. Cytokine-based high log-scale expansion of functional human dendritic cells from cord-blood CD34-positive cells. Sci Rep. (2011) 1:174. 10.1038/srep00174 22355689 PMC3240956

[38] Solito S Falisi E Diaz-Montero CM Doni A Pinton L Rosato A. A human promyelocytic-like population is responsible for the immune suppression mediated by myeloid-derived suppressor cells. Blood (2011) 118:2254–65. 10.1182/blood-2010-12-325753 21734236 PMC3709641

[39] Takeda N O'Dea EL Doedens A Kim JW Weidemann A Stockmann C. Differential activation and antagonistic function of HIF-{alpha} isoforms in macrophages are essential for NO homeostasis. Genes Dev (2010) 24:491–501. 10.1101/gad.1881410 20194441 PMC2827844

[40] Wang Z Cao L Kang R Yang M Liu L Zhao Y. Autophagy regulates myeloid cell differentiation by p62/SQSTM1-mediated degradation of PML-RARalpha oncoprotein. Autophagy (2011) 7:401–11. 21187718 10.4161/auto.7.4.14397 PMC3127220

[41] Parker KH Horn LA Ostrand-Rosenberg S. High-mobility group box protein 1 promotes the survival of myeloid-derived suppressor cells by inducing autophagy. J Leukoc Biol (2016) 100:463–70. 10.1189/jlb.3HI0715-305R 26864266 PMC4982609

