# Supplementary material for: GM-CSF Promotes the Expansion and Differentiation of Cord Blood Myeloid-Derived Suppressor Cells, Which Attenuate Xenogeneic Graft-vs.-Host Disease
Source: Front Immunol. 2019 Feb 26;10:183. doi: 10.3389/fimmu.2019.00183 (PMC6399310; doi:10.3389/fimmu.2019.00183)
Supplement: Supplementary file 1 [file Table_1.pdf]

# Supplementary Table 1.

## Antibodies for surface stain

| Antibodies | Fluorochromes | Clone names | Vendor | Catalog numbers |
|---|---|---|---|---|
| Anti-human CD33 | FITC | HIM3-4 | eBioscience | 11-0339 |
| Anti-human CD11b | PE | ICRF44 | eBioscience | 12-0118 |
| Anti-human HLA-DR | eFluor450 | L243 | eBioscience | 48-9952 |
| Anti-human CD14 | PE-Cy7 | 61D3 | eBioscience | 25-0149 |
| Anti-human CD15 | APC | MMA | eBioscience | 17-0158 |
| Anti-human CD11c | AF700 | 3.9 | eBioscience | 56-0116 |

| Antibodies | Fluorochromes | Clone names | Vendor | Catalog numbers |
|---|---|---|---|---|
| Anti-human CD3 | PE-Cy7 | UCHT1 | eBioscience | 25-0038 |
| Anti-human CD19 | FITC | 4G7 | BioLegend | 392508 |
| Anti-human CD56 | PE | TULY56 | eBioscience | 12-0566 |
| Anti-human CD34 | AF700 | 581 | BD Biosciences | 561440 |

| Antibodies | Fluorochromes | Clone names | Vendor | Catalog numbers |
|---|---|---|---|---|
| Anti-human HLA-ABC | FITC | W6/32 | eBioscience | 11-9983 |
| Anti-human CD80 | PE-Cy5 | 2D10.4 | eBioscience | 15-0809 |
| Anti-human CD86 | PE | IT2.2 | eBioscience | 12-0869 |
| Anti-human CD83 | APC | HB15e | eBioscience | 17-0839 |
| Anti-human CD40 | APC-H7 | 5C3 | BD Biosciences | 561211 |

| Antibodies | Fluorochromes | Clone names | Vendor | Catalog numbers |
|---|---|---|---|---|
| Anti-human CCR7 | FITC | 3D12 | eBioscience | 11-1979 |
| Anti-human CD13 | PE | WM-15 | eBioscience | 12-0138 |
| Anti-human CCR2 | PerCP-Cy5.5 | K036C2 | BioLegend | 357204 |
| Anti-human CXCR4 (CD184) | PE-Cy7 | 12G5 | eBioscience | 25-9999 |
| Anti-human ICAM-1 (CD54) | APC | HA58 | eBioscience | 17-0549 |
| Anti-human CD45 | AF700 | 2D1 | eBioscience | 56-9459 |

| Antibodies | Fluorochromes | Clone names | Vendor | Catalog numbers |
|---|---|---|---|---|
| Anti-human CCR5 | PE | eBioT21/8 | eBioscience | 12-1957 |
| Anti-human E-Cadherin (CD324) | AF488 | DECMA-1 | eBioscience | 53-3249 |
| Anti-human CD62L | APC-eFluor780 | DREG-56 | eBioscience | 47-0621 |
| Anti-human PD-L1 (CD274) | PE-Cy7 | MIH1 | eBioscience | 25-5983 |

# Supplementary Table 1.

## Antibodies for stain of regulatory T cells (Treg cells)

| Antibodies | Fluorochromes | Clone names | Vendor | Catalog numbers |
|---|---|---|---|---|
| Anti-human FoxP3 (intracellular) | PE | PCH10 | eBioscience | 12-4776 |
| Anti-human CD3 (surface) | PE-Cy7 | UCHT1 | eBioscience | 25-0038 |
| Anti-human CD4 (surface) | FITC | OKT4 | eBioscience | 11-0048 |
| Anti-human CD25 (surface) | APC | BC96 | eBioscience | 17-0259 |

## Antibodies for stain of Th1(IFN-γ), Th2 (IL-4), and Th17 (IL-17A) cells

| Antibodies | Fluorochromes | Clone names | Vendor | Catalog numbers |
|---|---|---|---|---|
| Anti-human CD3 (surface) | PE-Cy7 | UCHT1 | eBioscience | 25-0038 |
| Anti-human CD4 (surface) | FITC | OKT4 | eBioscience | 11-0048 |
| Anti-human IFN-γ (intracellular) | Pacific Blue | 4S.B3 | BioLegend | 502522 |
| Anti-human IL-4 (intracellular) | APC-Cy7 | MP4-25D2 | BioLegend | 500834 |
| Anti-human IL-17A(intracellular) | PerCP-Cy5.5 | eBio64DEC17 | eBioscience | 45-7179 |

## Antibodies for intracellular stain of immune suppressive molecules

| Antibodies | Fluorochromes | Clone names | Vendor | Catalog numbers |
|---|---|---|---|---|
| Anti-human iNOS2 | FITC | C-11 | Santa Cruz Biotechnology | SC-7271 |
| Anti-human IDO | PE | 700838 | R&D Systems | IC6030P |
| Anti-human Arginase1 | PerCP-Cy5.5 | 658922 | R&D Systems | IC8026C |

| Antibodies | Fluorochromes | Clone names | Vendor | Catalog numbers |
|---|---|---|---|---|
| Anti-human MPO | FITC | 8E6 | eBioscience | 11-1299 |

## Antibodies for stain of phosphorylated-(p)Stat1, pStat3, pStat6

| Antibodies | Fluorochromes | Clone names | Vendor | Catalog numbers |
|---|---|---|---|---|
| Anti-human pStat1 | AF647 | pY701 | BD Biosciences | 612597 |
| Anti-human pStat3 | PE | pY705 | BD Biosciences | 612569 |
| Anti-human pStat6 | PerCP-Cy5.5 | pY641 | BD Biosciences | 561195 |

## Antibodies for stain of phosphorylated-(p)mTOR, pAkt

| Antibodies | Fluorochromes | Clone names | Vendor | Catalog numbers |
|---|---|---|---|---|
| Anti-human pAkt | PE | pS473 | BD Biosciences | 560378 |
| Anti-human pmTOR | PerCP-efluor710 | MRRBY | eBioscience | 46-9718 |
